# The Association of Bullying and Suicidality: Does it Affect the Pediatric Population?

**DOI:** 10.7759/cureus.9691

**Published:** 2020-08-12

**Authors:** Therese Limbana, Farah Khan, Noha Eskander, Mina Emamy, Nusrat Jahan

**Affiliations:** 1 Psychiatry, California Institute of Behavioural Neurosciences and Psychology, Fairfield, USA; 2 Research, California Institute of Behavioural Neurosciences and Psychology, Fairfield, USA; 3 Internal Medicine, California Institute of Behavioural Neurosciences and Psychology, Fairfield, USA

**Keywords:** bullying, suicide, suicide psychology, bullying and suicide, bullying and suicide psychology

## Abstract

Over the last few years, bullying has been identified as one of the significant issues in the pediatric population. Reports also found that bullied youth have a higher risk of developing suicidality. Although preventable, suicide remains the leading cause of death in young people. This literature review aims to establish the association of bullying and the suicidality of the pediatric group (0-18 years of age). A PubMed search was conducted to find studies associating bullying and suicidality in the pediatric population. MeSH keyword strategy, along with subheadings, was used to retrieve appropriate literature. A total of 42 articles were included after the careful examination and application of exclusion and inclusion criteria. This study showed a strong association between bullying and suicidality, albeit the presence of some contradictory ideas.

## Introduction and background

The children and adolescent populations are generally considered to be healthier members of society both mentally and physically. However, over the last decade, reports of mental health problems have slowly increased in pediatric populations. Bullying is one of the significant issues identified. Bullying is a type of deliberate, repeated act of aggression, verbal or non-verbal, which involves a power difference from the victim to the perpetrator [1]. One of the newer forms of harassment is cyberbullying. It is associated with frequent use of the internet, text messaging, and threatening victims through the web [2].

More attention has been paid in the last few years to associating bullying and suicidality. Research studies have also made an effort to prove the link between these two entities [3]. One of the studies showed that children and teens victimized by perpetrators have been prone to suicide risk as they show more attempts to kill themselves and present with psychological problems [4]. It is common to think that only the victims of bullying have suffered mental health consequences. However, cyberbullying studies show that it is not only the cyber victim who suffers mental, psychosomatic, and social challenges but also the cyberbullies and the bystanders who observe the situation [2]. So, this fact means that finding ways to set up a bully-free environment is essential. 

As the mode of education slowly began shifting from a traditional classroom set-up to an online platform, young learners became more exposed to the virtual world. Parents, teachers, and mental health practitioners should be mindful of the experience of each of these children and adolescents encounter [2]. One of the critical factors that protect the individual’s ability to cope with stressful situations is emotional intelligence (EI). There is still an unestablished relationship between EI and suicide risk correlation in bully-victims even there are multiple studies conducted about it. It will be a breakthrough once a link is established, as the promotion of emotional skills would be a key driver in the development of academic and suicide prevention programs [5].

The prevalence of bullying victimization varies across the nations, but the fact that bullying leads to mental health problems and suicidal ideations is very consistent [6]. This study aims to establish an association between harassment and the propensity of children and young people (0-18 years of age) to commit suicide. If bullying means derangement to the mental health of the children and adolescents, then prioritizing a bully-free environment should have the highest priority [7].

Method

In PubMed, literature was selected based on MeSH keywords with corresponding subheadings. Table 1 shows MeSH keywords in a literature search.

**Table 1:** MeSH keywords for literature search

MeSH Keywords:	
Bullying	
Total Records	4104
Records Selected	2678
Suicide (Subheading-Psychology)	
Total Records	20058
Records Selected	7213
Bullying and Suicide (Subheading-Psychology)	
Total Records	118
Records Selected	100

The following inclusion and exclusion criteria were used during the literature search:

Inclusion Criteria:

Human studies

Child: birth- 18 years

Published papers in the English language

All abstracts and full papers

Exclusion Criteria:

Animal studies

Non- English literature

Table 2 shows in order the total number of articles according to the inclusion and exclusion criteria.

**Table 2:** The total number of articles according to the inclusion and exclusion criteria

MeSH Keywords:	
Bullying	
Total Records	4104
Human population	4047
English literature	3947
Child (0-18 years old)	2765
Records Selected	2678
Suicide (Subheading-Psychology)	
Total Records	20058
Human population	20001
English literature	17753
Child (0-18 years old)	7970
Records Selected	7213
Bullying and Suicide (Subheading-Psychology)	
Total Records	118
Human population	118
English literature	111
Child (0-18 years old)	106
Records Selected	100

Results

Following a refined search of the MeSH keyword ‘Bullying and Suicide (Subheading-Psychology),’ 100 abstracts, free full-texts, and full-text articles were attained. Of the 100 articles reviewed, 56 were excluded from the reasons set below:

Not indicating the association between Bullying and Suicidality

Inability to gain access to paid articles

Meta-analysis studies

**Figure 1:**
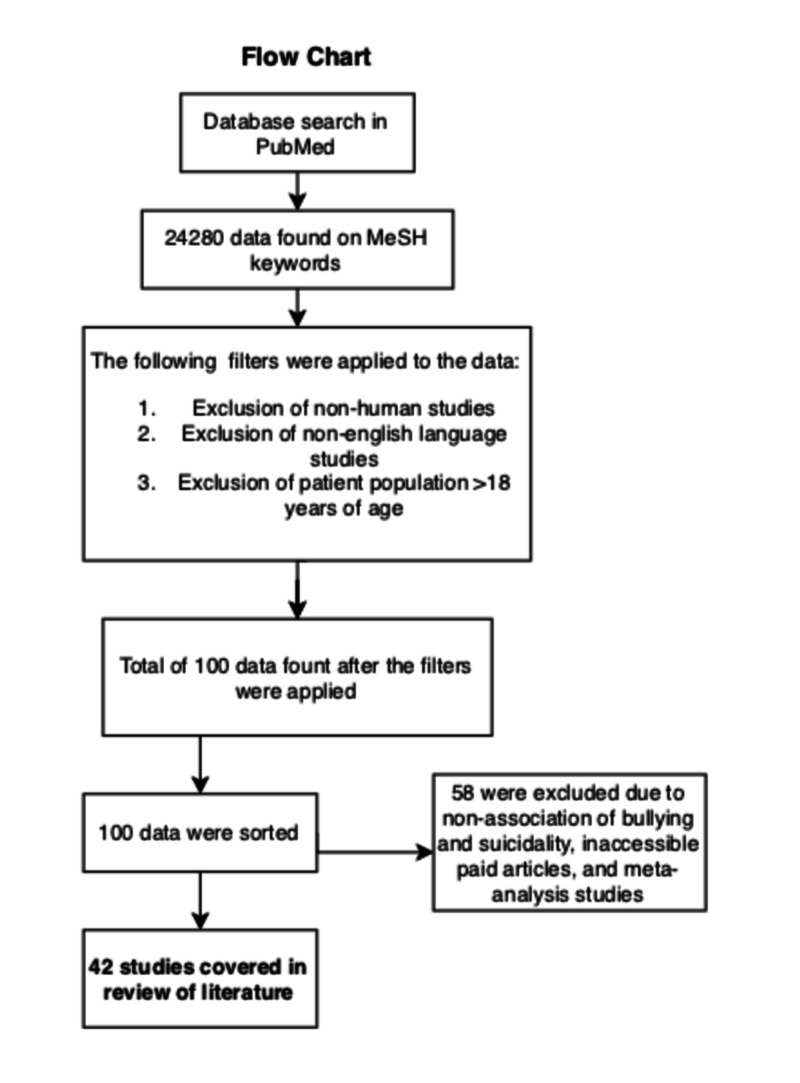
Flow chart of the literature review process

Figure 1 describes the process of the current literature review.

## Review

Bullying and suicide

The third most common cause of death among 10-24 years old is suicide. Although it is preventable, it still is on the top spot on the chart [8]. Upon looking at suicide statistics, there is an elevated trend of suicidal thoughts to 13-year-old teens and suicidal attempts to 15-year-old teens at 11.6%-14.7% and 5.4%-6.8%. Compared to the teens outside of these age groups, there is a significantly low percentage of reports at 2.7%-4.1% for suicidal thoughts, and 1.6-1.9% for suicidal attempts were noted [4]. The number of perceived life adversities seemed to favor the relationship towards youth suicidality, according to some key findings of a systematic review [9]. In Greece, where reports of suicide are deficient, a cross-sectional study was conducted wherein late adolescents revealed that their lives are no longer worthwhile after experiencing bullying situations. Bullying and suicidality have a complex association when there is a difference in victim and perpetrator risks involving confounding variables, and when the bullying-suicide relationship is not going toward a predictable direction [10]. Another cross-sectional study was conducted in Boston in 2008 among high school students. It was reported that those involved in bullying were at risk of suicidal ideation and attempts with the highest threat to victim-perpetrators [11,12]. High school teens are identified to have more psychological issues and are also more vulnerable to stress due to the transitioning process they encounter when they become high schoolers [13]. That is why this group needs to be carefully guided and provided with open communication channels anytime they need help [14]. Victims also showed a recurrence of self-injury thoughts and behaviors [15]. A prospective study of 206 American boys from a high-crime community was categorized as victims, bully, or bully-victims. Later in life, the consequences of how they were classified in the victim-bully spectrum were assessed. It showed that all of the three groups developed depression [16]. Suicide attempts, tobacco use, and several arrests are common to bully-victims [15,16]. And among the perpetrator group, violent arrests and tobacco-use were found [16].

Bullying is often classified into two forms, namely, physical and non-physical. Either way may increase the tendency of youth to suicidality and other psychiatric illnesses [1]. In recent times, children are challenged with another type of bullying subclassified under non-physical, known as cyberbullying [7,17]. A cross-sectional study in Arkansas shows that out of all affected school-goers, 11.6% were bullied in school, 6.2% were cyberbullied, and 10.2% experienced both forms [18]. Cyberbullying has led the parents and teachers to be concerned about how our youth are exposed to violence online through web media. Its prevalence is around 6.55-35.9%, according to studies [2]. American middle school surveys have shown that this type has triggered more depression and suicidality than any conventional bullying [17]. There are also instances reported that merely being an onlooker to bullying activities, either online or physically, already increases the individual's mental health risk [19].

Today, more researchers have focused their attention on studying about bullying and its consequences to save the young people and to stop the traditional thinking that bullying is just a part of growing up. Perpetrators in bullying are not only their peers but also the adult group. Victims with adult perpetrators have shown higher health-risk behaviors, including worse illicit-substance use and suicidal attempts [20]. In a case-control study of bullied young people at 9, 11, and 13 years of age, it was observed that after seven years from the bullying incident, they were likely to develop anxiety and panic disorders [21]. Bullying is a danger to children and youth. It may not immediately cause suicidality, but enormous effects on the children's mental and physical health are alarming. Youth often believed that after encountering a difficult situation, they feel unwanted by friends, they struggle initiating relationships, and they undergo extreme loneliness. Other adolescents feel tensed and have unexplained abdominal pains [21].

Mediators of bullying

Several variables affect the relationship between bullying and suicidality. In a case-control study conducted to 67 adolescents at Midwestern Children Hospital, negative self-esteem worsens the tendency to commit suicide [22]. Depression, anxiety, loneliness, and helplessness are factors that were also acknowledged to aggravate the situation [3,23]. It is noted that substance abuse, unprotected sex, and violence have intensified the suicidal predilection in physical and cyber-bullying [24]. There is nothing more important than to raise awareness of the risks of bullying and change the idea that this is an acceptable part of childhood experience [25].

As humans, we are social beings, and it is essential to connect with other people to express our thoughts and feelings. As social beings, the degree of connection to friends and family affects how teens react to bullying events [26]. Social exclusion is noted as one of the top factors that influence mental health and suicidality [7]. A good teacher-student relationship may protect youth from the horrible effects of the event [27]. Parental support has a moderating influence on adolescents' mental health but does not help much on homophobic bullied groups. It just shows that mental health workers should be a part of the picture to help out parents to provide the right support to this vulnerable group [28]. The use of force or being coerced should also be considered as a risk factor. Students who are compelled to do a sexual act, forced to stay in an abusive relationship, or threatened with any weapon were found to have a higher likelihood of developing suicidality [29]. They are also twice as likely to develop depression and contemplated suicide than those who have not encountered these intimidating events [29]. There are psychiatric issues in the background of bullying. These conditions should be taken into consideration during assessment and treatment to address the main problem [30]. It is essential to educate health-care workers the approaches in dealing with bullying situations and the suicide risk assessment tools they may use to timely address the case [8]. Some studies would also point out that although many mental health problem factors contribute to bullying, these psychiatric issues are not the only reason for it. Perpetrators may inflict violence or harm on the basis that they want to do it. So, it was suggested that perpetrators undergo behavioral therapies and psychiatric counseling and face legal actions [1].

All over the world, bullying and suicidality is a reality that affects children not just in the United States. School-bullying was rampant in South Korea in the past decades, but after the government's efforts, an improvement started to get noticed, such as fewer reports of bullying in recent years [17]. Reports from Israel, Lithuania, Luxembourg, Latin-America [6], and across the continents consistently show how disastrous bullying involvement can be [31]. Exposure to the country's political violence intensifies the primary problem [32]. These youth groups, living in a geographical location who always experience war, should be protected, and strategies must be developed to help them. Bullying is indeed a serious matter that needs to be addressed without wasting time to reduce future risks [33]. The longer the exposure to the threat, the higher the chance of suicidality [4]. This problem needs full cooperation by agencies, schools, and as well as by families and friends of individuals in this highly vulnerable group.

Emotional intelligence

In a cross-sectional study conducted to 4533 adolescents using the Chinese version of the School Bullying Experience Questionnaire, risk and protective factors vary among adolescents depending on the extent of how the individual youth perceived the initial bullying experience [34]. Although these experiences have a severe impact on these individuals' futures, good mental health developed during childhood may cushion them from its harmful effects [35]. Rey et al. (2019) would show that good emotional intelligence (EI) reduces suicide risk [9]. Another cross-sectional study supports the idea that a higher EI is a modifying factor protecting youth from developing suicidal tendencies [5]. It means that the higher EI an individual has, the higher the chance one can have to overcome devastating situations [9].

Teen minority groups

Since part of the teens' population are sexual minority youth (SMY) groups such as the lesbian-gay-bisexual-transgender (LGBT) community and the female population, studies were done to assess the effects of bullying specifically in this group. In a cross-sectional study by Bouris et al. (2016), they found that SMY groups experience higher suicide risk compared to an ordinary youth individual [36]. LGBT childhood sexual abuse in men and sexual with physical violence in women has contributed to the increasing incidence of suicide attempts [37]. Williams et al. (2017) also found that female populations have more verbal and social bullying reports, while the male population reports more physical bullying [14]. Additive interaction of bully victimization and depressive symptoms affected mostly the female population [38]. The primary key point to remember is that it is essential to monitor bullying reports (verbal and non-verbal) to recognize the problem and address self-inflicted injuries and suicidality immediately [39]. An environment that would protect and ensure SMY groups' safety should also be considered, especially in a school set-up [40].

Table 3 shows some of the studies with results that would prove the association of bullying and suicidality.

**Table 3:** Cross-sectional studies with a positive association of bullying and suicidality

Author/Publication Date	Study design	Sample size	Main Points
Borowsky et al. [25], 2013	Cross-sectional study	130,908	All perpetrators (22%), victims (29%), and bully-victims (38%) stated to have experienced suicidal thoughts and attempts; factors related to a higher risk of suicidality to perpetrators only and victims only; strong parental-bond and presence of friends were reported to be protective factors
Geoffroy et al. [4], 2016	Cross-sectional study	1,168	Youth experiencing peer victimization have higher suicidal ideation risk at 13 years of age, while suicide attempts were reported in 15 years old population; prolonged exposure to victimization, the more chances of developing suicidal thoughts, and mental health issues
Sigurdson et al. [33], 2018	Cross-sectional study	2,464	Bullied youths presented with self-injury, suicidal thoughts, and attempted suicide; female participants reported reduced suicidal thoughts as they got older, while male participants had higher suicidal attempts

Negative association

Although there is a strong correlation of bullying to suicidality of the youth population, there are still reports that would not agree with the simple cause and effect relationship between the two entities. These studies believe that bullying behavior is not a stand-alone factor that leads to suicide [19,41]. There are still so many factors that could influence the individual to suicidality. In the developmental victimology framework, heightened mental health risks contributed to many forms of victimization. Examples such as lack of supervision to the kids, unstable family relationships, and personal characteristics are the few factors. All of these may lead to issues that affect the mind of the youth [42]. One prospective cohort study that monitors the high-school students involved in bullying showed that only those with baseline suicidal risk behaviors had been shown to have internalized responses four years after the initial assessment [41].

Table 4 presents studies supporting the negative association of bullying and suicidality.

**Table 4:** Studies with a negative association between bullying and suicidality results

Author/Publication Date	Study design	Sample size	Main Points
Klomek et al. [41], 2011	Cohort study	236	Participation in any bullying activities without other risk factors did not indicate subsequent depression, suicidal attempts, or ideation; after four years since the study was conducted, internalization issues were noted more on students who had baseline suicidal risk behaviors than those who had bullying behaviors; results were firm in pointing out that after a two-year follow-up among Australian youth, victimization did not predict the current mental health status of the subjects
Sinyor et al. [19], 2014	Cross-sectional study	94	Bullying is an uncommon component that leads to suicide; Depression (51.3%) and parental conflict (27%) were identified as circumstances leading to suicide; bullying resulted in 6 deaths (8.1%), and it was the only contributing factor in 5 deaths; over 14 years only six suicide deaths were reported despite the 2006 report that 10% of the youth in Canada experience bullying; criminal, school-related, and romantic relationship issues were more common to lead to death than bullying; youth suicide is a result of several intricate interactions

Multiple factors could lead to the suicidality of the youth. Bullying is recognized as one of the most critical issues that cause the increasing incidence of suicidality and suicide deaths based on reports. With this in mind, research studies that measure the probability of suicidality to troubled youth are suggested. Bullying does not only affect youth groups but as well as the other population. More research studies are also recommended, especially for older people who are already at the twilight of their years. The present literature review has limitations that should be taken into consideration. The study restricts its scope in terms of age (0-18 years old), language (English language studies only), and only human studies were covered. All types of research designs were included except for meta-analysis, which was excluded in this literature review.

## Conclusions

This study aims to determine the relationship between bullying and its propensity to develop suicide ideation, attempts, and plans among the children and adolescent population. This study established a strong correlation between the two entities. Bullying does not just increase the risk of the youth to suicidality. It also intensifies the likelihood of developing depression, anxiety, and post-traumatic stress disorders, to name a few. It is not only the victims that suffer from bullying, but perpetrators have also heightened the risk of developing psychiatric issues. Bullying is controllable, and suicidality is highly preventable. As this is so, society should give full attention and develop strategies to implement a bully-less, if not a bully-free environment to these highly vulnerable groups. Most studies collated had a limitation in following the sample population in a prolonged period, limiting the subjects' chronological and successive observation. It is highly recommended for future research to conduct a longitudinally designed study. This highly at-risk group is our future. Protecting their mental-health and providing a safe and nurturing environment should be a priority.
